# Temperature dependence of phonon-defect interactions: phonon scattering vs. phonon trapping

**DOI:** 10.1038/srep32150

**Published:** 2016-08-18

**Authors:** M. B. Bebek, C. M. Stanley, T. M. Gibbons, S. K. Estreicher

**Affiliations:** 1 Physics Department, Texas Tech University, Lubbock TX 79409-1051, USA

## Abstract

The interactions between thermal phonons and defects are conventionally described as scattering processes, an idea proposed almost a century ago. In this contribution, ab-initio molecular-dynamics simulations provide atomic-level insight into the nature of these interactions. The defect is the Si|X interface in a nanowire containing a δ-layer (X is C or Ge). The phonon-defect interactions are temperature dependent and involve the trapping of phonons for meaningful lengths of time in defect-related, localized, vibrational modes. No phonon scattering occurs and the momentum of the phonons released by the defect is unrelated to the momentum of the phonons that generated the excitation. The results are extended to the interactions involving only bulk phonons and to phonon-defect interactions at high temperatures. These do resemble scattering since phonon trapping occurs for a length of time short enough for the momentum of the incoming phonon to be conserved.

## Introduction

The transport of energy through materials occurs via charge carriers (usually electrons) and atomic vibrations (thermal phonons). The phonon contribution involves the out-of-equilibrium coupling of the normal vibrational modes of the system. It is well known that the presence of defects reduces the flow of thermal phonons. ‘Defect’ refers here to any disruption to the perfect crystal: impurities, grain boundaries, interfaces, surfaces, etc. The present contribution is a first-principles study of phonon-defect interactions. They involve the coupling between delocalized (bulk-related) and localized (defect-related) vibrational modes.

### The origin of Phonon Scattering

The first description of phonon-defect interactions was published by Peierls^1^ in 1929. A few years later, the word ‘phonon’ was invented^2^ but it would not become widely used for another two decades. Peierls visualized propagating ‘lattice waves’ and thought of defects as (static) perturbations to the perfect crystal. He proposed that they scatter lattice waves. A few years later, Casimir^3^ argued that surfaces also reduce heat flow by scattering such waves. It was not fully recognized at the time that defects profoundly affect the properties of the material. Defect physics as a field of research emerged only after World War II with the advent semiconductors.

By the 1960s, lattice-wave scattering had become phonon scattering. This was theoretically refined by Klemens^4^, Ziman^5^, and Callaway^6^ and scattering lifetimes were associated with the interactions between host-crystal phonons – normal (n) and Umklapp (U) scattering – as well as between thermal phonons and grain boundaries (gb), impurities (i), and other defects. The phonon lifetime τ is obtained from Matthiessen’s rule 1/τ = 1/τ_n_ + 1/τ_U_ + 1/τ_gb_ + 1/τ_i_ + … This description ignores the dynamic properties of defects. Phonon-defect scattering has become the accepted way to think. Such interactions preserve the momentum of the incoming phonon and are often assumed to be elastic as well (see ref. 7 for a recent review). Some authors argued^8^ that the defect’s degrees of freedom should not be ignored, but the calculation of normal vibrational modes for a defect embedded in a crystal involved dynamical matrices far too large to be computationally tractable at that time.

### Phonon trapping

Decades of experimental and theoretical studies of defects in semiconductors have shown that defects are associated with low- and high-frequency vibrational modes. Many of them are observed by infra-red absorption or Raman spectroscopy^9^, or produce phonon sidebands in photoluminescence spectra^10^. Transient-bleaching spectroscopy studies^11^,^12^,^13^ have shown that the excitations of high-frequency impurity-related modes survive for substantial lengths of time and often exhibit unusually large isotope effects^13^,^14^. These results have been explained^13^,^14^,^15^,^16^ with ab-initio MD simulations using the ‘supercell preparation’ scheme (see below).

A compilation of measured vibrational lifetimes vs. the estimated order of the decay, known as the ‘frequency-gap law’^17^, has shown that one-phonon processes (resonant coupling) occur within a few tenths of a ps, while two- and three-phonon processes involve 5–10 ps and over 80 ps, respectively. Similar times scales are expected for both the decay and the excitation of vibrational modes with frequency ω such that ħω > k_B_T_0_, where T_0_ is the background temperature.

Ab-initio molecular-dynamics (MD) studies of thermal phonon-defect interactions^18^,^19^,^20^ have highlighted the key ingredients involved. First, defects introduce vibrational modes that are localized in space at or near the defect (Spatially-Localized Modes or SLMs). Second, the vibrational lifetimes of (high- and low-frequency) SLMs are much longer than those of bulk modes: SLM excitations survive for dozens (sometimes hundreds) of periods of oscillations while bulk modes typically decay within about one period: Localized modes do not couple easily to delocalized ones. Thus, defects trap small amounts of energy in SLMs for meaningful lengths of time, a phenomenon called ‘phonon trapping’. This suggests that defects reduce heat flow – at least in part – because of phonon trapping.

These studies have shown that phonon trapping occurs, but the contribution of phonon scattering has not been discussed. Defect-related SLMs exist in specific frequency ranges. Thermal phonons with the right frequency (i.e. at the right temperature) quickly excite these SLMs by resonant coupling. But what happens to thermal phonons in temperature ranges where the defect exhibits no SLMs? Does one deal with phonon scattering complemented by phonon trapping? Which of these interactions dominates? The ab-initio MD simulations discussed here imply that phonon-defect interactions involve *only* phonon trapping and that the momentum of the phonons released by the defect is unrelated to that of the thermal phonon that created the excitation.

The present study involves a δ-layer in a Si nanowire. The defect is the Si|X interface where X is C (lighter than Si) or Ge (heavier than Si). On one side we have Si-Si bonds, on the other side X-X bonds, and the ‘interface’ as narrowly defined here consists only of the atoms involved in Si-X bonds. There are several reasons for choosing such a system. First, heterojunctions are ubiquitous in semiconductor technology: oxide or surface layers, heavily-implanted regions, δ-layers, superlattices, and other such structures are very common. Second, the interface is highly localized in the direction of heat flow and the SLMs associated with it are well defined. Third, the theoretical description of this nanostructure is quite simple and the geometry can be fully optimized using the periodic cluster approach described below. Fourth, the interactions between heat flow and interfaces have been the subject of numerous studies at various levels of theory and are a topic of interest. However, we have studied the thermal properties of many impurities and defects, and the coupling between SLMs and thermal phonons does not depend on the nature of the defect that generates the SLMs.

### Empirical studies of heat flow and interfaces

Theoretical studies of the impact of an interface on heat flow started with the acoustic mismatch model (AMM)^21^. It was introduced to explain the thermal resistance at the solid-superfluid He interface observed by Kapitza^22^. AMM uses the acoustical impedances of the materials on both sides of the interface to obtain the thermal conductance. The model was later used to interpret the thermal transport at solid-solid interfaces, but only gave consistent results at very low temperatures^23^. It also failed to predict the observed boundary resistance for grain boundaries since there is no difference between the acoustic impedances on both sides of the interface.

The diffuse mismatch model (DMM)^24^ assumes that all the incoming phonons are elastically scattered at the interface and that the materials on both sides of the interface are isotropic. The thermal coefficients of transmission and reflection are determined by the phonon densities of states of the materials. DMM produces results that are qualitatively consistent with experiment but often over- or under-estimates the reflection and transmission coefficients^25^. Both the AMM and DMM models ignore the degrees of freedom associated with the interface itself and the thermal conductance is independent of the temperature.

An atomistic approach using lattice dynamics in the harmonic approximation has also been proposed^26^. A limited numbers of nearest neighbors are assumed to be interacting. The phonon density of states is obtained using uniformly distributed k-points and the group velocities are calculated from the phonon dispersion curves. The heat flow rate from one material to the other is calculated considering only the phonons travelling at normal incidence to the interface. Empirical potentials are used to describe the interatomic interactions^27^ but the role of the interface modes is not discussed.

Another method based on dynamical matrices involves Green’s functions^28^,^29^,^30^. The dynamical properties of each material are obtained as if they were not in contact^31^ and the interface is introduced as an off-diagonal correction. The eigenvectors are then written in terms of those of the perfect materials plus the Green’s function associated with the correction. In order to obtain the Kapitza conductance, the energy flux across the interface is calculated and then divided by the temperature difference between two heat reservoirs.

Many studies of heat flow at interfaces involve empirical MD simulations. No assumption is made about the nature of the thermal transport. In thermal equilibrium^32^,^33^,^34^,^35^,^36^,^37^, the system has a constant average temperature and the average heat flux is zero, but a finite heat flux is caused by fluctuations. The decay of the fluctuations of the interfacial energy flux is recorded and interfacial resistance (or conductance) is obtained via the fluctuation-dissipation theorem.

In many empirical non-equilibrium MD simulation, the system is prepared in a steady state by creating a temperature gradient using two heat reservoirs (thermostats) on both sides of the interface which is normally placed halfway between the two reservoirs. Reducing the amplitude of the temperature fluctuations requires extensive thermalization runs until a steady-state is reached^38^,^39^,^40^,^41^. The temperature drop across the interface and the known heat flux generated by the thermostats are used to find the interface thermal conductance. This approach does not provide any detail about the atomistic interactions between thermal phonons and the interface.

Finally, phonon wave-packet dynamics has been used to study phonon scattering at interfaces^39^,^42^,^43^,^44^. A wave packet is constructed from a Gaussian distribution of a narrow range of phonons from an acoustic branch of the phonon dispersion curve. Empirical MD simulations are performed, the wave packet propagates towards the interface, and the reflected and transmitted components are analyzed. An application of the method for Si with a SiO_2_ layer shows that a small part of the energy (3%) is trapped in the layer, and then slowly dissipates into the bulk Si material on either side of the layer^43^. The existence of interface-related modes has been reported by other authors^44^ who argued that these modes may facilitate the transfer of the energy by serving as a bridge for inelastic interactions and explain the measured temperature dependence of the thermal interface conductance^45^. This analysis is generally consistent with the predictions of ab-initio MD simulations^18^.

## The Theoretical Challenge

Electronic-structure calculations involving defects are best handled using first-principles theory rather than semi-empirical methods^46^. Indeed, the values of parameters fitted to an experimental database are not easily transferable to defects which often exhibit strained configurations distinct from those to which the parameters have been fitted. ‘First-principle’ refers to density-functional theory combined with ab-initio MD simulations. There are no parameters fitted to experimental values. The quantitative nature of the results predicted by this combination of tools is abundantly documented by decades of studies of defects in semiconductors. Such calculations are now common-place in periodic supercells containing several hundred atoms, but they are computationally demanding when extensive MD runs are required.

A complication arises from the non-equilibrium MD runs. Indeed, the usual way to initiate such calculations involves a Maxwell-Boltzmann distribution of nuclear velocities corresponding to the temperature T_0_ while all the nuclei are in their equilibrium (minimum-energy) sites at the time t = 0. This does produce the desired temperature, but the MD run begins with all the vibrational modes exactly in phase (zero potential energy), an unphysical situation. At first, all the nuclei move together, losing and gaining kinetic energy simultaneously. As a result, the initial temperature fluctuations are comparable to T_0_ and must be reduced with a thermostat and extensive thermalization runs until the system reaches a reasonable steady-state. This situation makes it impossible to study phonon coupling. Indeed, the temperature fluctuations are larger than many phonon energies and the vibrational modes couple to the thermostat much faster (every few MD time steps) than to each other.

The solution is an approach called supercell preparation. It involves random distributions of individual mode phases and energies at the time t = 0. No thermostat is used, the temperature fluctuations are very small starting with MD step 1 and, more importantly, they remain almost perfectly constant with time. The details of the technique have been published^20^ and only the key points need to be summarized here.

## First-Principles MD Without Thermostat

The electronic structures are obtained from first-principles calculations: Norm-conserving ab-initio-type pseudopotentials for the electronic core regions and density-functional theory for the valence regions. Any density-functional software package can be employed. We use the SIESTA method^47^,^48^, in which the valence states are described with numerical pseudo-atomic basis sets: double-zeta for elements of the first two rows of the Periodic Table with polarization functions for 3^rd^-row elements. The orbital radii for the surface atoms have been optimized using the approach proposed by Garcia-Gil *et al*.^49^,^50^. We use the local-density approximation in calculations involving only light elements and a revised generalized-gradient approximation^51^ when heavier elements are involved. The geometries are optimized using conjugate-gradients and the dynamical matrix is calculated.

**Figure 1:**
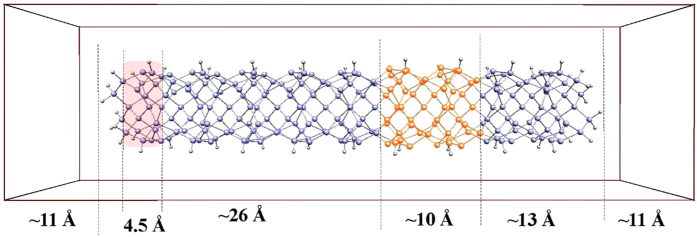
Si_184_Ge_50_H_56_ nanowire in its 1D-periodic box. Each nanowire is separated from its nearest image by ~22 Å of vacuum. The nanowire starts in thermal equilibrium at the temperature T_0_ and a gradient is introduced by warming a slice (reddish box on the left) of the nanowire to T_hot_.

The host material for most of our calculations is Si_184_X_50_H_56_, a H-terminated Si nanowire containing a δ-layer of atoms X, where X is Ge or C. This nanowire is placed in a 1D-periodic ‘box’ of dimensions larger than the nanowire. Each end of the nanowire is separated from its nearest image by about 22 Å of vacuum (Fig. 1). This construction has two advantages. First, when running non-equilibrium MD simulations, there is no thermal contamination between image nanowires: each cluster is strictly microcanonical. Second, once a temperature gradient is set up by heating one end of the nanowire, heat flows initially in just one direction. The time step is about 1/40^th^ of the fastest oscillation, typically 1 fs.

Our method^20^ differs from other MD simulations in three important ways. First, no empirical potentials are used. Second, we do not set up and maintain a temperature gradient or perform thermalization runs until a steady-state is established. Instead, we set up a T gradient at the time t = 0 and then monitor the system as it returns to equilibrium without using a thermostat: the normal vibrational modes couple only to each other. Third, we minimize the T fluctuations by averaging over many initial microstates (see below).

The key ingredient is the dynamical matrix which is calculated at T = 0 K. The orthonormal eigenvectors e_αi_^s^ give the relative displacement of atom α along i = x, y, z for each mode s. These eigenvectors are related to the Cartesian coordinates r_αi_ via the normal-mode coordinate q_s_, r_αi_(T, t) = Σ_s_ e_αi_^s^ q_s_(T, t)/√m_α_ where T is the temperature and t the time. Even though the MD runs are fully anharmonic with forces obtained from the total energies, the conversion between Cartesian and normal-mode coordinates involves an assumption for the unknown q_s_. We use q_s_(T, t) = A_s_(T)cos(ω_s_t + φ_s_). This introduces a random distribution of phases at the time t = 0.

The amplitudes A_s_(T) are obtained from the condition that, in thermal equilibrium, the average energy per mode is k_B_T, where k_B_ is the Boltzmann constant. We use a randomized Boltzmann distribution of energies β exp{−βE_s_}, where β = 1/k_B_T_0_, which produces the average energy per mode k_B_T. Using the inverse-transform method for generating distributions, the cumulative distribution function ζ_s_ = ∫_0_^Es^ β exp(−βE) dE gives E_s_ = −k_B_T_0_ ln(1 − ζ_s_), where ζ_s_ is a random number in the interval [0, 1]. Equating this to the energy E_s_ = ½A_s_^2^ω_s_^2^, we get A_s_^2^(T_0_) = −2 k_B_T_0_ ln(1 − ζ_s_)/ω_s_^2^. An initial microstate is one specific distribution of modes phases and energies at the time t = 0. Since there is an infinite number of initial microstates corresponding to the same macrostate, we must average the MD runs over n initial microstates. Averaging over n = 30 runs reduces the temperature fluctuations to the point where temperature changes of the order of 1 K or even smaller can be monitored. This allows us to use very small T gradients.

Note that the dynamical matrix is necessarily calculated at 0 K, and therefore are not exact at non-zero temperatures. Indeed, vibrational frequencies are known to slowly shift as T increases^9^, and therefore the eigenvectors of the dynamical matrix slightly change as well. Even though the normal-mode basis set is imperfect at non-zero T, it remains a very good approximation up to at least room temperature.

In order to study heat flow, a thin slice of the cell is prepared at a higher temperature (T_hot_) than the rest of the cell (T_0_). The modes are kept in phase at the interface by preparing the entire cell at T_0_ and then increasing the mode amplitudes until the atoms in the warmer slice are at T_hot_^20^.

In order to calculate the lifetime of a vibrational mode with frequency ω > k_B_T_0_/ħ, the system is prepared at T_0_ for all the modes except the one of interest which is assigned 3ħω/2 potential energy (zero-point energy plus one phonon). This classical oscillator then has the same initial amplitude as the quantum-mechanical one. During the MD run, the Cartesian coordinates at every time step are converted into normal-mode coordinates, which allows us to monitor the energy and amplitude of all the modes vs. time. Note that the added potential energy shifts the temperature from T_0_ to T_cell_ since 3Nk_B_T_cell_ = (3N−1)k_B_T_0_ + 3ħω/2, where N is the number of atoms^20^.

## Temperature Dependence of Phonon-Defect Interactions

We now use the interface-related SLMs to predict the behavior of a heat front at a Si|Ge or Si|C interface. Then, we perform ab-initio MD simulations and record the temperature of the interface and of the δ-layer vs. time. Since SLMs exist in specific frequency ranges, the predictions depend on the background temperature and that of the heat front as it reaches the interface. The predictions account only for phonon trapping and the subsequent decay of the excitation. Unpredicted changes in temperature would be associated with phonon scattering.

**Figure 2:**
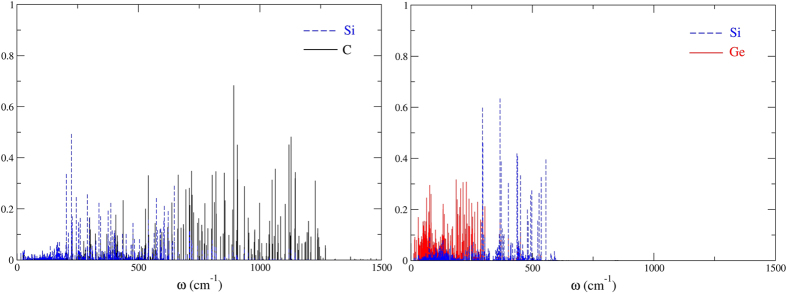
Left: Localization L_α_^2^(ω) of the SLMs associated with the Si|C interface. The sum over α includes all the Si (blue) and C (black) atoms involved in Si-C bonds. Right: Same plot for the Si|Ge interface, with the Ge contributions in red.

The interface SLMs are obtained from the orthonormal eigenvectors e_αi_^s^ of the dynamical matrix. Since they are normalized, the sum over all the atoms Σ_α_L_α_^2^ = Σ_α_ (e_αx_^s^)^2^ + (e_αy_^s^)^2^ + (e_αz_^s^)^2^ = 1. The localization^52^ of mode s on just one atom or a small group of atoms α is Σ_α_L_α_^2^ < 1. The plot of L_α_^2^(ω) shows the localization of all the modes on the atom(s) α. Figure 2 show all the SLMs associated with the Si|C and Si|Ge interfaces.

One prominent feature is that the Si|C interface exhibits no SLMs below ~200 cm^−1^ while the Si|Ge interface has many Ge-related SLMs in that frequency range. Two temperatures play a role: T_0_, the background temperature and T_hf_, the temperature of the heat front as it reaches the interface. We determine the time when the heat front reaches the location of the interface from MD simulations made under the same temperature conditions but without the δ-layer. T_hf_ is the temperature of a thin Si layer just before the interface at that time. The equilibrium temperature T_eq_ is the final temperature the nanowire would reach given long enough simulation times. But our simulations are too short for the entire nanowire to reach thermal equilibrium.

**Figure 3:**
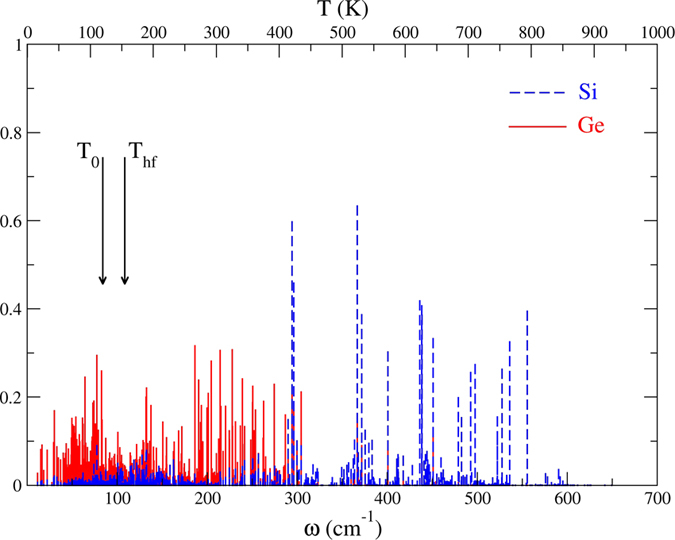
Distribution of SLMs in the low-frequency region of the Si|Ge interface. The background and heat front temperatures are T_0_ = 120 K and T_hf_ = 155 K, respectively.

**Figure 4:**
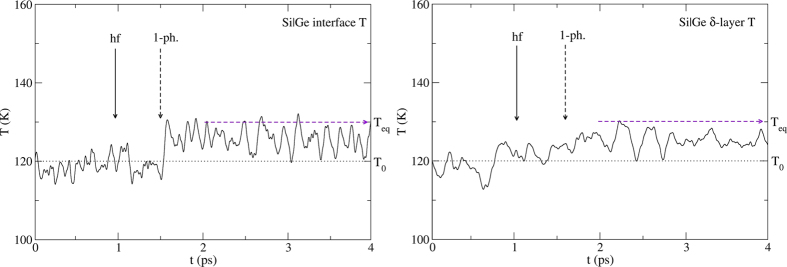
Left: Temperature of the interface vs. time obtained from ab-initio MD simulations. The heat front reaches the interface at t = 1 ps and the temperature begins to rise at t~1.5 ps, a delay compatible with phonon trapping (resonant excitation) as well as with phonon scattering. Right: Calculated temperature of the Ge δ-layer. Its temperature begins to increase at about the same time as the interface because the interface SLMs also involve the motion of a few atoms in the Ge layer. The dashed horizontal line shows the initial temperature T_0_ = 120 K. The horizontal arrow shows the final (equilibrium) temperature of the entire nanowire T_eq_ = 130 K. The plots involve a 50 time-steps running average. The MD runs are averaged over about 50 initial microstates.

We consider first the Si|Ge interface (Fig. 3) with T_0_ = 120 K and produce a hot slice on the Si side such that T_hf_ = 155 K. There are numerous SLMs in the temperature window 120–155 K and they should be resonantly excited (one-phonon process) by the heat front. We therefore expect that phonon trapping by the SLMs will cause the temperature of the interface to increase a few tenths of a ps after the heat front reaches it (about 1 ps at that temperature, Fig. 4, left). We also expect the δ-layer to pick up some temperature almost simultaneously (Fig. 4, right). Indeed, the SLMs are not 100% localized at the interface but involve the motion of some Ge atoms in the layer as well. The result of our ab-initio MD simulations confirm these expectations. But the result is very much the same one expects from phonon scattering interactions which involve similar time scales.

**Figure 5:**
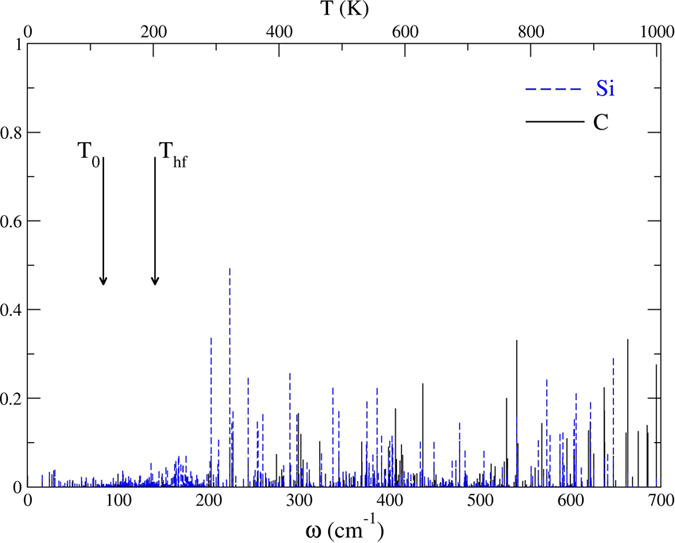
Distribution of SLMs in the low-frequency region of the Si|C interface. The background and heat front temperature are T_0_ = 120 K and T_hf_ = 200 K, respectively.

**Figure 6:**
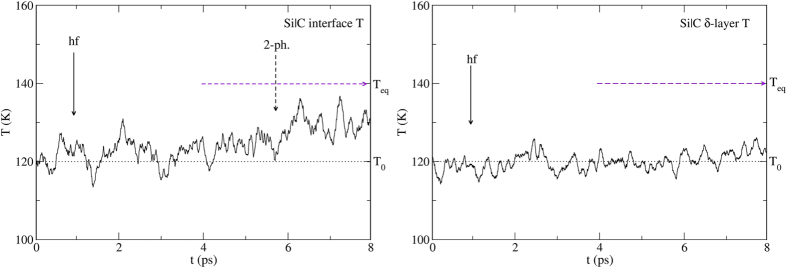
Left: The heat front reaches the interface at t = 1 ps but the temperature begins to rise only after about t~5–6 ps. This delay is caused by phonon trapping since two-phonon processes are required, but it is not compatible with phonon scattering since momentum-conserving interactions should involve a few tenths of a ps. Right: The temperature of the C δ-layer remains constant and its temperature is not expected to increase until at least 12 ps. But phonon scattering should result in at least some fast increase in the temperature of the layer. The dashed horizontal line shows the initial temperature T_0_ = 120 K. The horizontal arrow shows the final (equilibrium) temperature of the entire nanowire T_eq_ = 140 K. The plots involve a 50 time-steps running average. The MD runs are averaged over about 50 initial microstates.

We now use T_0_ = 120 K and T_hf_ = 200 K for the Si|C system (Fig. 5). There are no interface-related SLMs in this temperature window and therefore no phonon trapping occurs by resonant excitation. If we assume that only phonon trapping is at play, we should have to wait ~5 to 6 ps for two-phonon processes to excite higher-frequency SLMs before the temperature of the interface increases. Figure 6 left, confirms that this is indeed what happens. And then, the temperature of the δ-layer can only increase after the excited interface SLMs decay, but since the C-C modes have even higher frequencies than the interface Si-C ones, this again requires two-phonon processes. Thus, phonon trapping and decay should lead to an increase in the temperature of the δ-layer only after 10 to 12 ps, a longer simulation time than we can achieve here. Any faster increase in the temperature of the δ-layer would have to be associated with processes other than phonon trapping, such as phonon scattering. But the MD simulations show no such increase (Fig. 6, right). This rules out any contribution to heat flow from processes in which the momentum of the incoming phonons is conserved. The interactions are entirely determined by phonon trapping and decay. The knowledge of the interface SLMs suffices to determine the outcome.

**Figure 7:**
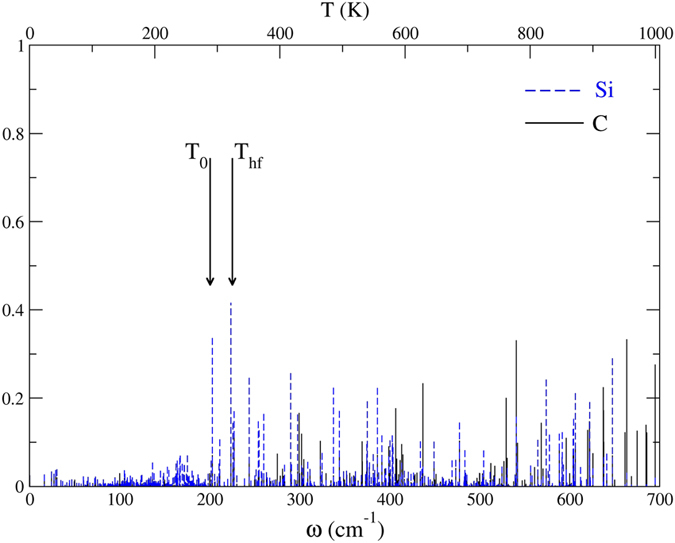
Distribution of SLMs in a frequency region of the Si|C interface that includes SLMs. The background and heat front temperatures are T_0_ = 280 K and T_hf_ = 315 K, respectively.

**Figure 8:**
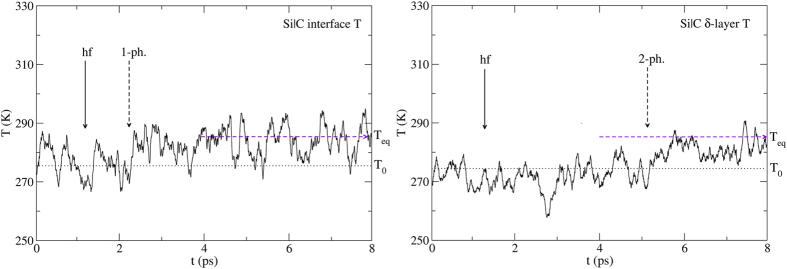
Left: Temperature of the interface vs. time obtained from ab-initio MD simulations. The temperature begins to rise after 2 ps, shortly after the heat front reaches the interface. Right: The temperature of the δ-layer remains T_0_ for almost 6 ps, as two-phonon processes are needed to excite the higher-frequency C-C modes. Phonon scattering would lead to a much faster increase in the temperature of the layer. But this does not occur. The dashed horizontal line shows the initial temperature T_0_ = 280 K. The horizontal arrow shows the final (equilibrium) temperature T_eq_ = 290 K. The plots involve a 75 time-steps running average. The MD runs are averaged over 25 initial microstates.

Finally, we change the background and heat front temperatures to match the presence of SLMs at the Si|C interface: T_0_ = 280 K and T_hf_ = 315 K (Fig. 7). This narrow temperature window should result in a rapid increase of the interface temperature. Since the thermal conductivity of the Si nanowire is higher at 280 K than at 120 K^50^,^53^, the heat front reaches the interface after about 0.5 ps. As expected, the interface temperature readily increases (Fig. 8, left). But then, in order for the δ-layer to gain energy, one must wait for the excited interface Si-C modes to decay into the higher-frequency C-C modes, and this involves two-phonon processes. This is precisely what the MD simulations show (Fig. 8, right). Here again, the behavior is entirely dictated by phonon trapping. No rapid increase in temperature of the δ-layer that could be assigned to phonon scattering occurs.

These results are not limited to two specific interfaces in this Si nanowire. First, SLMs with long vibrational lifetimes are present in all the defects we have studied. They include a variety of point defects (isolated impurities, isotope substitutions, small complexes) as well as surfaces, and various interfaces^14^,^15^,^16^,^18^,^19^,^20^. All of these SLMs can trap phonons for many periods of oscillation (this is also observed experimentally^11^,^12^,^13^). Second, ab-initio MD simulations in the 1D-periodic supercell Si_225_X_25_H_40_ (X is Ge or C) at 120 K show a qualitatively identical effect with larger T gradients than the ones used here^19^,^20^. Third, a study of the impact of the surface of the nanowire at the same level of theory^50^ has shown that thermal phonons do not scatter off the surface. The excited Si-H surface modes couple to each other very efficiently (resonant coupling, a one-phonon process) rather than decay into Si modes (a much slower two-phonon process). Finally, the ‘bulk’ Si modes rarely excite surface modes, because this requires two-phonon processes. The coupling of surface and bulk modes is highly inefficient. Instead, most of the heat propagates fast through the bulk (without coupling to the surface) and the rest of the heat propagates slowly on the surface (without coupling to the bulk). Reaching thermal equilibrium requires waiting for both bulk and surface contributions. Fourth, calculations of the thermal conductivity in the presence of impurities^20^ also shows phonon trapping as the mechanism that reduces heat flow, not phonon scattering.

Note that our calculations include all the normal vibrational modes of the system. Since this nanostructure is quite small, only a small number of low-frequency, often called long-wavelength, phonons are included (they have a wavelength longer than the size of the interface). Calculations that include a large number of long wavelength phonons are planned at the empirical level since they require hundreds of thousands of atoms.

## Key Points and Extension to Normal Scattering

Ab-initio MD simulations of heat-flow interacting with a Si|Ge or Si|C interface have been performed without thermostat. The use of small temperature gradients allows us to focus on different parts of the vibrational spectrum of the interfaces, where SLMs are present or absent. These SLMs must be excited by the incoming heat front before the temperature of the δ-layer can increase. The decay of SLMs depends on the availability of receiving modes and not on the origin of the excitation. In other words, the momentum of the emitted phonons is unrelated to the momentum of the incoming phonons. A similar situation occurs in fluorescence, which involves photon trapping (albeit for a much longer time). If scattering implies that the momentum of the outgoing phonons is related to the momentum of the incoming phonon, then no phonon scattering by the defect occurs.

When the background and heat front temperatures correspond to frequency regions where interface SLM are present, phonon trapping involves resonant coupling (one-phonon process) and the temperature of the interface increases within a few tenths of a ps as seen in Fig. 4 (left) and 8 (left). The decay of the excitations into the δ-layer occurs just as quickly if receiving modes are available in the required frequency range (Fig. 4, right) but requires two-phonon processes (Fig. 8, right) when no such receiving modes are present. On the other hand, when the background and heat front temperatures correspond to frequency regions where no interface SLM are present, then phonon trapping itself is delayed as it requires two- (or higher) phonon processes (Fig. 6, left) and the decay into the δ-layer is delayed even further (Fig. 6, right). No temperature increase of the δ-layer occurs until the excitations in the interface SLM decay into it. This rules out any ‘interface scattering’ event since it would always increase the temperature of the δ-layer within a few tenths of ps.

This situation is qualitatively comparable to gas-phase molecular collisions. Two types of reactions are observed^54^. In a *direct* reaction such as D + CH_4_ → HD + CH_3_, the scattering is *not isotropic* and the reaction conserves the momentum of the incoming ion. Such a reaction involves a fraction of a ps. In an *indirect* reaction such as Cl^−^ + CH_3_Br → CH_3_Cl + Br^−^, the reactants collide, remain associated in an intermediate ion-molecule complex (in this example: Cl^−^–CH_3_Br), and then the products scatter *isotropically*. A time delay of several ps is associated with the rotation of the molecule until the relevant bonding orbitals are oriented along a direction that makes the reaction possible. But then the momentum of the reactants is no longer related to the momentum of the incoming ion. The time scales involved are the same as those associated with the one- and two-phonon processes discussed above.

What can be said about the interactions involving only bulk (delocalized) phonons, the so-called normal scattering processes? (In the vicinity of a defect, the periodicity is lost, the Brillouin zone and Umklapp processes are no longer defined). Do these interactions conserve the momentum of the incoming phonon, consistent with a scattering event? We need to extend an argument involving the coupling between delocalized (bulk-related) and localized (defect-related) vibrational modes to only delocalized modes. We start with a point defect with strongly localized SLMs and a long vibrational lifetime which obviously involves phonon trapping: substitutional C in Si. This defect is characterized by four short C–Si bonds, a strong perturbation to the crystal. We move on to substitutional Ge (a much weaker perturbation), then to ^30^Si in ^28^Si, and finally to ^29^Si in ^28^Si, a very weak perturbation indeed. As the perturbation associated with the substitutional impurity X becomes weaker, we expect the vibrational lifetime of the SLMs to become shorter. We calculated the SLMs and vibrational lifetimes at 75 K in the ^28^Si_63_X periodic supercell.

The dominant SLM for substitutional ^12^C in ^28^Si is at 597 cm^−1^ (measured: 607 cm^−1^ 55). It is ~79% localized on the C atom and its calculated vibrational lifetime is τ = 12.2 ps (240 periods of oscillation). In the case of ^74^Ge, the SLM at 104 cm^−1^ is only 21% localized with τ = 3.9 ps (12 periods). For ^30^Si and ^28^Si in ^28^Si, the SLMs at 538 cm^−1^ and 539 cm^−1^ have τ = 0.13 ps (2.1 periods) and τ = 0.09 ps (1.5 periods), respectively.

We still deal with phonon trapping, but for shorter and shorter lengths of time, until τ is so short that it becomes comparable to the vibrational lifetime of bulk modes (see Table 1 in ref. 19). We started with highly-localized SLMs with long vibrational lifetimes and end up with much more delocalized modes and vibrational lifetimes so short that the momentum of the incoming phonon is largely conserved. In terms of the chemical analogy above, we moved from an ‘indirect’ to a ‘direct’ reaction. Thus, when only delocalized vibrational modes are involved, phonon trapping occurs for such short lengths of time that the distinction between trapping and scattering becomes blurred.

A very similar situation occurs at high temperatures, where the meaning of ‘high’ depends on the SLMs. Indeed, the lifetime of SLM excitations decreases with temperature^11^,^12^. When these lifetimes drop to a period of oscillation or less, phonon trapping occurs for such a short time that phonon-defect interactions become similar to scattering processes. Therefore, the physical processes involved in phonon-defect interactions are best studied at low temperatures with carefully selected temperature windows.

Finally, we comment on the Kapitza resistance associated with the interface between materials A and B: Is it related to phonon trapping? The answer is probably ‘no’ because the Kapitza resistance involves time scales much longer than the vibrational lifetimes of individual SLMs. However, it could be the consequence of the decay of the SLM excitations. Indeed, this decay depends on the availability of receiving modes in the materials A and B at the particular temperature of the system. In some temperature ranges, heat will bounce back into material A because no receiving modes are available in material B. This results in a second and then a third round of phonon trapping leading to a hotter interface. In other temperature ranges, the balance of receiving modes changes, most excited SLMs decay rapidly into material B, and the interface appears colder. This qualitative argument requires detailed investigations but if it is correct, then the Kapitza resistance should be temperature dependent for a given pair of materials. Experiments involving small temperature gradients are needed.

We fully recognize that the present arguments are based on the results of ab-initio MD simulations while the truth lies in the hands of clever experimentalists. Measuring temperatures in tiny regions (an interface) at the ps time scale is very challenging. Simpler experiments could involve measuring how long it takes for a system to reach thermal equilibrium under identical (small) excitations but with different background temperatures. For example, a Si sample with a C layer and a ~50 K excitation at T_0_ = 100 K, 300 K, or 500 K would return to equilibrium after different lengths of time relative to the defect-free material with the same temperature gradients. At the lowest T, the SLM excitations and their decay in the C layer both require two-phonon processes. At very low T, they may even require three-phonon processes. At a higher T, phonon trapping by the interface occurs resonantly but the decay requires two-phonon processes. At still higher T, both trapping and decay involve much faster one-phonon processes. Such predictions require only the knowledge of the dynamic properties of the interface – the central ingredient of phonon-defect interactions.

## Additional Information

**How to cite this article**: Bebek, M. B. *et al*. Temperature dependence of phonon-defect interactions: phonon scattering vs. phonon trapping. *Sci. Rep.* **6**, 32150; doi: 10.1038/srep32150 (2016).
